# Six Weeks of Morning Fasting Causes Little Adaptation of Metabolic or Appetite Responses to Feeding in Adults with Obesity

**DOI:** 10.1002/oby.22452

**Published:** 2019-03-29

**Authors:** Enhad A. Chowdhury, Judith D. Richardson, Javier T. Gonzalez, Kostas Tsintzas, Dylan Thompson, James A. Betts

**Affiliations:** ^1^ Department for Health University of Bath Bath UK; ^2^ School of Life Sciences Queen’s Medical Centre, University of Nottingham Nottingham UK

## Abstract

**Objective:**

The aim of this study was to determine the effects of sustained morning fasting or breakfast consumption on metabolism, energy intake, and appetite in healthy adults with obesity.

**Methods:**

An independent‐measures randomized controlled trial with baseline and follow‐up laboratory assessment days separated by a 6‐week intervention of either morning fasting (0 kcal until 12:00 pm) or daily breakfast (> 700 kcal by 11:00 am) was performed. Measures included metabolic outcomes (glucose, insulin, nonesterified fatty acids), hormones regulating appetite (total/acylated ghrelin, peptide YY, leptin), and energy expenditure (diet‐induced thermogenesis) parameters throughout a laboratory test day and *ad libitum* intake following a fixed breakfast.

**Results:**

Allocation to fasting versus breakfast resulted in minimal adaptation as reflected by the metabolic outcomes or the majority of appetite regulatory outcomes for either area under curve or time‐course‐based measures (*P* > 0.05). *Ad libitum *lunch intake was not different (*P* = 0.13), nor was diet‐induced thermogenesis or a composite appetite score (both *P* > 0.10). However, there was a reduced total area under the curve for peptide YY (*P* = 0.05) and increased postprandial hunger ratings (*P* = 0.05) in the breakfast group.

**Conclusions:**

There was little evidence of metabolic adaptation to acute feeding or negative consequences from sustained morning fasting. This indicates that previously observed differences between breakfast consumers and skippers may be acute effects of feeding or may have resulted from other lifestyle factors.

## Introduction

There is a strong public perception that breakfast consumption is beneficial for weight management 1. Consistent with this point of view, less frequent or insufficient breakfast consumption is associated with a greater risk for excess weight in adults 2, 3, 4. Evidence regarding energy intake from cross‐sectional studies has been divided, with some indicating similar total intake between breakfast consumers and skippers 4, 5 and others greater intake among those consuming breakfast 6, 7. Further work from the National Health and Nutrition Examination Survey demonstrated greater energy intake on days that breakfast was reported within the same individuals 8. Despite these disparities within the observational research, much evidence from randomized trials has suggested that skipping breakfast reduces overall energy intake 9, with this effect apparent in both free‐living 10, 11, 12 and laboratory contexts 13, 14, 15, 16.

It was reported that self‐reported habitual breakfast consumption can affect acute responses to morning fasting or feeding 17, with the authors suggesting this finding may be due to metabolic and appetite regulatory system entrainment. However, it is unknown whether these effects are due to breakfast habits per se or other positive health behaviors displayed by breakfast consumers. Therefore, the causal effects of regular breakfast consumption on acute appetite and metabolic responses to morning feeding in individuals with obesity remain unknown.

We previously reported that regular breakfast consumption did not causally affect acute metabolic or hormonal responses to feeding in lean individuals 18. However, individuals with obesity have been shown to display unique responses to feeding, including delayed satiation 19, reduced concentrations of satiety hormones 20, and limited suppression of ghrelin with feeding 21, 22. Beyond these differences, our group has also observed differing responses to chronic feeding interventions from the most fundamental level of molecular adaptation 23 to free‐living energy intake between lean individuals 10 and individuals with obesity 24. Therefore, there is a need to characterize the chronic adaptation to regular morning fasting versus breakfast consumption on acute appetite, metabolic, and energy intake responses to feeding in individuals with obesity.

The present study aimed to investigate for the first time whether sustained (6‐week) exposure to either a morning fasting or daily breakfast regimen can cause adaptation of metabolic responses, acute appetite regulation, and energy intake at an *ad libitum *lunch in individuals with obesity. Based on the results from our work in lean individuals, we hypothesized that there would be no adaptation of acute appetite and metabolic responses to feeding by either daily morning fasting or breakfast consumption.

## Methods

### Participants

Twenty‐two men (*n* = 8) and women (*n* = 14) with obesity aged 25 to 58 years took part in this study. Participants were recruited via local advertisement from South West England and were initially assessed for eligibility based on BMI ≥30 kg/m^2 ^and then later classified as having obesity based on dual‐energy x‐ray–derived fat mass indices of ≥9 kg/m^2^ (men) and ≥13 kg/m^2^ (women) 25. Written informed consent was obtained from all participants, with the ethical approval for the study obtained from the National Health Service South West 3 Research Ethics Committee (10/H0106/13). The study was conducted in accordance with the ethical standards in the 1964 Declaration of Helsinki and its later amendments.

This study was part of a larger program of work titled the Bath Breakfast Project (ISRCTN31521726), for which the full project protocol has been previously published 26. The sample size for this study was based on the estimates for the wider project. This was based on the number of individuals in each treatment group (~14) required to confer a 90% probability to detect a 646‐kcal increase in physical activity energy expenditure during the free‐living component of the study with use of a 2‐tailed *t* test with an α level of 0.05. In accordance with eligibility criteria previously set out for this population 24, participants reported adhering to a standard sleep‐wake cycle (e.g., no shift workers) and not anticipating any change in lifestyle during the study period. Participants were free of metabolic disorders, with premenopausal female participants either menstruating regularly or following their chosen contraceptive method (e.g., pill, implant) for >6 months. Participants were required to report being weight stable (±2% body mass fluctuation within past 6 months). Characteristics of participants are presented in Table 1.

**Table 1:** Participant characteristics

	Breakfast group	Fasting group
***n***	11	11
**Age (y)**	43 (10)	44 (10)
**Body mass (kg)**	104.0 (24.0)	92.4 (11.2)
**BMI (kg/m^2^)**	35.0 (6.1)	32.0 (2.3)
**Fat mass index (kg/m^2^)** a		
**All**	14.8 (5.0)	12.0 (2.3)
**Female**	16.9 (4.5)	13.2 (1.8)
**Male**	9.9 (1.4)	9.8 (0.8)
**Resting metabolic rate (kcal/d)**	1,679 (335)	1,613 (257)
**Fasting glucose (mmol/L)**	5.3 (0.8)	5.3 (0.4)
**Fasting insulin (pmol/L)**	71 (39)	50 (18)
**Habitual breakfast consumers (*n*)** b	7	6
**Female (*n*)**	7	7

Values shown as mean (SD). No statistically significant differences between groups at baseline.

a Fat mass index calculated as dual‐energy x‐ray–derived total fat mass divided by height squared. Dual‐energy x‐ray–derived fat mass index obesity ranges (25) = ≥13 kg/m^2^ (women) and ≥9 kg/m^2^ (men).

b Defined as ingestion of ≥50 kcal within 2 hours of waking on most days of the week.

### Study design

**Figure 1:**
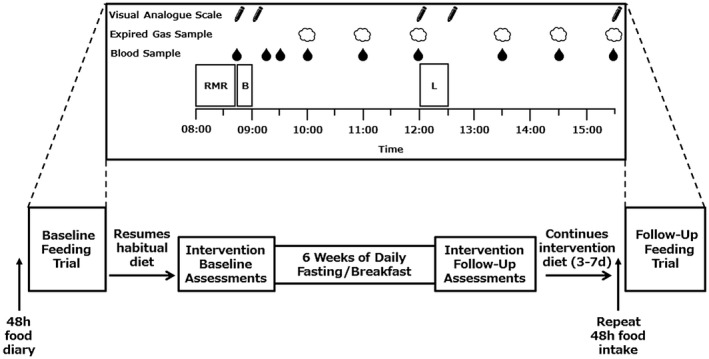
Study design schematic. In menstruating women, the intervention commenced 2 weeks after the baseline assessments so that the intervention follow‐up visit was conducted in the follicular phase of the cycle (3‐10 days after onset of menses). There were some cases in which the first breakfast feeding trial occurred after the baseline assessment but before the intervention beginning, so it was also in the follicular phase. The 48‐hour food diary completed before the first feeding trial and repeated before the follow‐up feeding trial conformed to the assigned intervention of the participants. The top half of the figure shows the protocol during the feeding trials. B, breakfast; L, *ad libitum *lunch; RMR, resting metabolic rate.

The Bath Breakfast Project involved 3 sequential phases undertaken by the same individuals in cohorts of lean individuals and those with obesity 26. These were (1) a crossover laboratory experiment contrasting the acute metabolic and/or appetite responses to breakfast versus fasting on a given day 15, 16, (2) a free‐living component contrasting the chronic behavioral and metabolic responses during 6 weeks of daily breakfast versus morning fasting 10, 24, and (3) an examination of the acute metabolic and/or appetite responses to feeding following the aforementioned intervention period 18 (Figure 1). This allows us to examine whether sustained adherence to a given morning feeding pattern can result in a chronic adaptation in the acute response to daily meals.

### Intervention

During the 6‐week interventions previously described in full 10, 24, participants were randomized (1:1 allocation ratio) into either a group prescribed an energy intake of ≥700 kcal before 11:00 am daily, with at least half consumed within 2 hours of waking (Breakfast Group) or a group abstaining from energy‐providing nutrients until 12:00 pm each day (Fasting Group). For those consuming breakfast, there were no stipulations on the composition of morning intake. Beyond the morning period, all other lifestyle choices were allowed to vary naturally. The randomization of participants was stratified according to baseline breakfast habits to control for the distribution of habitual breakfast consumers and nonconsumers in each group.

### Standardization of participants prior to second feeding trial

Between the 2 laboratory visits for feeding trials, participants undertook their assigned dietary intervention for 6 weeks with pre‐ and postintervention visits to assess body composition changes and glycemic control (oral glucose tolerance test) 24. Following the postintervention visit, within 3 to 7 days, participants returned for their second breakfast feeding trial. During this intervening period, participants continued to follow their assigned intervention (i.e., ≥ 700‐kcal energy intake by 11:00 am or 0 kcal until 12:00 pm daily), such that any chronic adaptations to their intervention were not affected by a change of dietary practices before their follow‐up breakfast trial. As a result, the total adherence to the participants’ assigned intervention diet was 6 to 7 weeks. Participants completed 48 hours of diet standardization that corresponded to their assigned free‐living intervention and refrained from strenuous physical activity before both of the feeding trials.

### Protocol for laboratory visits

The experimental protocol (Figure 1) is identical to the breakfast feeding trials outlined in previous studies from this program of work 15, 16 and analytical procedures as described in full in our protocol paper 26. In brief, participants reported to the laboratory at 8:00 am ± 1 hour, when adherence to standardization measures were confirmed verbally. Participants then voided and had body mass measured in light clothing (Seca 873, Vogel and Halke, Hamburg, Germany). As a repetition of participants’ first visit to the laboratory prior to undertaking their assigned intervention, resting metabolic rate (RMR) was assessed according to best practice suggestions for participant preparation and measurement conditions 27. A cannula was then inserted into an antecubital vein, with a baseline sample obtained. Participants then consumed breakfast, with blood samples taken at 15 minutes, 30 minutes, and 1 hour post completion of the breakfast. One hour after breakfast, an expired gas sample was also obtained for assessment of diet‐induced thermogenesis and substrate oxidation. Blood and gas samples were obtained hourly until 3 hours post breakfast, at which point an *ad libitum* lunch was provided. Upon completion of the lunch period (30 minutes), samples of blood and expired gas were obtained each hour for another 3 hours. Participants also completed visual analogue scales relating to appetite at selected time points throughout the day. During the day, participants remained sedentary and completed quiet activities such as reading, watching television, and typing.

### Breakfast

The breakfast consisted of Corn Flakes (Kellogg’s, Battle Creek, Michigan), 2% fat milk, toasted white bread, margarine, and fresh orange juice provided in the same proportions for all individuals. Participants were given the choice of either white sugar added to cereal, seedless raspberry jam on their toast, or an isocaloric combination of both. The percentages of energy from macronutrients were 69.6% carbohydrate, 17.5% fat, and 12.9% protein. Breakfast was provided in quantities that contained 0.06 g carbohydrate/kcal of each individual participant’s measured daily RMR at baseline, resulting in energy intake of 525 ± 106 kcal and 537 ± 80 kcal in the breakfast and fasting groups, respectively. Participants were first provided with cereal, and then toast and orange juice at 5‐minute intervals, with the breakfast consumed within 15 minutes to standardize effects of eating rate on appetite hormones 28.

### *Ad libitum* lunch

The *ad libitum *lunch test meal consisted of cooked penne pasta and tomato sauce prepared at a ratio of 1:1 uncooked mass. The overall percentages of energy from macronutrients were 79% carbohydrate, 14% fat, and 7% protein. During the first trial, participants were permitted *ad libitum *intake of water during lunch; this volume was subsequently replicated on their second visit. Participants were alone during the lunch, with a recorded message played prior to consumption asking them to eat until they had satisfied their hunger. Pasta was provided in a large bowl containing 1 kg of cooked pasta and sauce, which was replenished every 10 minutes during the lunch period to minimize visual feedback and prevent any tendency to finish the portion provided. The mass of pasta consumed during the lunch was recorded, and energy intake was calculated using manufacturer’s nutritional information.

### Expired gas analysis

Five‐minute Douglas bags were employed to obtain expired air samples with concurrent measurement of inspired air composition 29. Relative proportions of oxygen and carbon dioxide were measured in a known volume of sample using paramagnetic and infrared analyzers, respectively (Servomex 1440, Crowborough, UK). Rates of both oxygen utilization (VO_2_) and carbon dioxide production (VCO_2_) were used to calculate energy expenditure as previously described 30, corrected for urinary nitrogen excretion 31, as follows:

energy expenditure = (3.941 × VO_2_) + (1.106 × VCO_2_) + (2.17 × nitrogen excretion).

### Blood sampling and analysis

Blood was collected and stored as serum or plasma using standard methods, apart from samples for analysis of acylated ghrelin, for which blood was treated with proteases to prevent degradation as previously described 15. Total ghrelin (intra‐assay coefficient of variation [CV], 4.0%) and acylated ghrelin (intra‐assay CV, 4.2%; Bertin Pharma, Montigny le Bretonneux, France) and peptide YY (PYY) (intra‐assay CV, 4.3%) assays were conducted using plasma. Leptin (intra‐assay CV, 3.4%) (R&D Systems Inc., Abingdon, UK) and insulin (intra‐assay CV, 4.7%) (Mercodia AB, Uppsala, Sweden) assays were conducted using serum. All samples were batch analyzed upon study completion and samples from each participant assayed on the same plate. Plasma samples were analyzed for nonesterified fatty acids (intra‐assay CV, < 5 %), glucose (intra‐assay CV, < 5 %), and urea (intra‐assay CV, < 5 %) using a Daytona automated analyzer (Randox Laboratories, Crumlin, Northern Ireland) according to manufacturer guidelines.

### Urine collection

Urine was collected in containers with 5 mL of 10% thymol isopropanol used as a preservative. The urine collected during a measurement period was mixed and a 1‐mL aliquot removed and stored at −80°C. Urinary urea concentrations for use in calculations to estimate urinary nitrogen excretion (and, hence, nonprotein respiratory exchange ratio) were obtained via immunoassay as described for plasma.

### Appetite sensations

Visual analogue scales were employed to assess subjective appetite. These scales were completed pre and post breakfast, pre and post lunch, and following a 3‐hour postprandial period after lunch. Participants marked responses to questions assessing desire to eat, hunger, fullness, and prospective consumption with anchor phrases on the ends of each of the scales (e.g., not at all hungry vs. as hungry as I have ever felt). Higher scores are indicative of greater sensations. A composite appetite score 32 was also calculated using the following formula: (desire to eat + hunger + [100 − fullness] + prospective consumption) / 4 to give an overall indication of appetite at the measured time points.

### Statistical analysis

For comparison of time series variables measured over the course of the day (e.g., appetite hormones), 3‐way mixed‐model ANCOVA was employed to identify interactions between group (i.e., breakfast vs. fasting), trial (i.e., baseline vs. follow‐up), and time point (i.e., time of day), all independent of deviations from a normal distribution 33 but with Greenhouse‐Geisser corrections applied to intraindividual contrasts for ɛ < 0.75 and the Huynh‐Feldt correction applied for less severe asphericity 34. Area under the curve (AUC) from the baseline trial was used as a covariate. Where relevant, to provide a fuller appreciation of any potential changes in response to the interventions, AUC as well as peak, nadir, and fasting concentrations specific to each participant (e.g., the mean of the peak concentrations within each participant, irrespective of when this occurred) was calculated, with these summary statistics subsequently analyzed by 2‐way ANCOVA, with baseline scores used as a covariate. Data are presented in the text as mean ± SD, and figures display mean with error bars representing SEM. All statistical analyses were conducted using SPSS Statistics version 22 (IBM Corp., Armonk, New York). Statistical significance was accepted at *P* ≤ 0.05.

## Results

### Mass and RMR

Body mass was relatively stable between the 2 trials, with both groups’ postintervention body mass on their second breakfast feeding trial within 0.9 kg of their preintervention body mass. RMR was 1,613 ± 257 kcal/d in the fasting group and 1,679 ± 335 kcal/d in the breakfast group prior to the intervention. In response to the interventions, RMR in the groups was stable within 10 kcal/d.

### Energy intake

**Figure 2:**
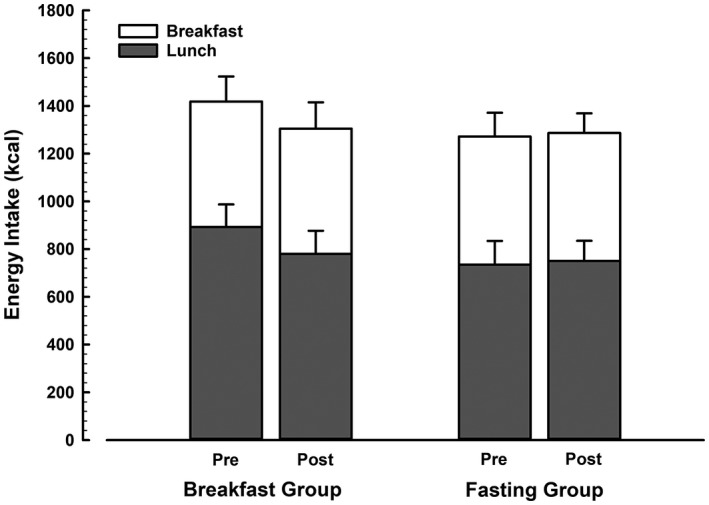
Energy intake during the feeding trials before and after 6 weeks of daily breakfast or fasting. Breakfast intake was prescribed, with the breakfast provided for both groups based on similar RMR (breakfast group, 525 ± 106 kcal vs. fasting group, 537 ± 80 kcal; *P* = 0.78) and did not change before to after the intervention. Error bars on the grey portion of the stack represent SEM of the energy intake at lunch, and error bars on the white portion represent SEM of the energy intake of the whole day.

There was no group × trial interaction for energy intake during the *ad libitum* lunch (*P* = 0.13). In the breakfast intervention group, lunch intake was reduced by 113 kcal (893 ± 313 vs. 779 ± 323) after the intervention. Lunch intake was stable (735 ± 330 kcal vs. 750 ± 284 kcal) before and after the intervention in the fasting group. Overall intake of breakfast and lunch in those consuming breakfast for 6 weeks was 1,418 ± 350 kcal prior to the intervention and 1,305 ± 366 kcal following the intervention. In those who underwent the fasting intervention, intake was 1,272 ± 331 kcal pre intervention and 1,286 ± 274 kcal post intervention (Figure 2).

### Systemic metabolites

**Figure 3:**
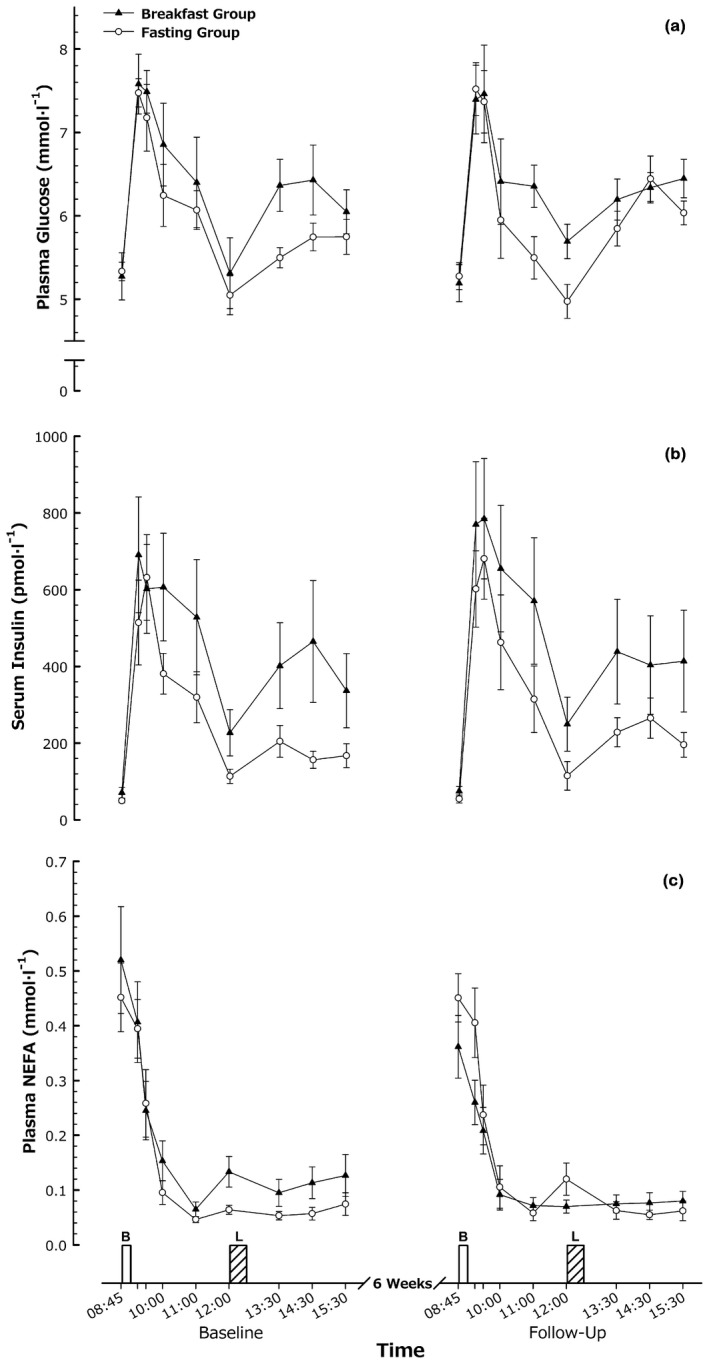
Metabolic responses. (**A**) Plasma glucose, (**B**) serum insulin, and (**C**) plasma nonesterified fatty acid (NEFA) responses to feeding in men and women with obesity before and after a 6‐week intervention. Values are mean ± SEM. B, breakfast; L, *ad libitum* lunch.

Concentrations of plasma glucose, nonesterified fatty acids, and serum insulin all varied throughout the day as would be expected (Figure 3). There were no group × trial interaction effects for any of the parameters (all *P* > 0.10). Comparisons of peak, nadir, or fasted concentrations of the metabolites did not display any group × trial interactions (all *P* ≥ 0.16).

### Energy balance regulatory hormones

**Figure 4:**
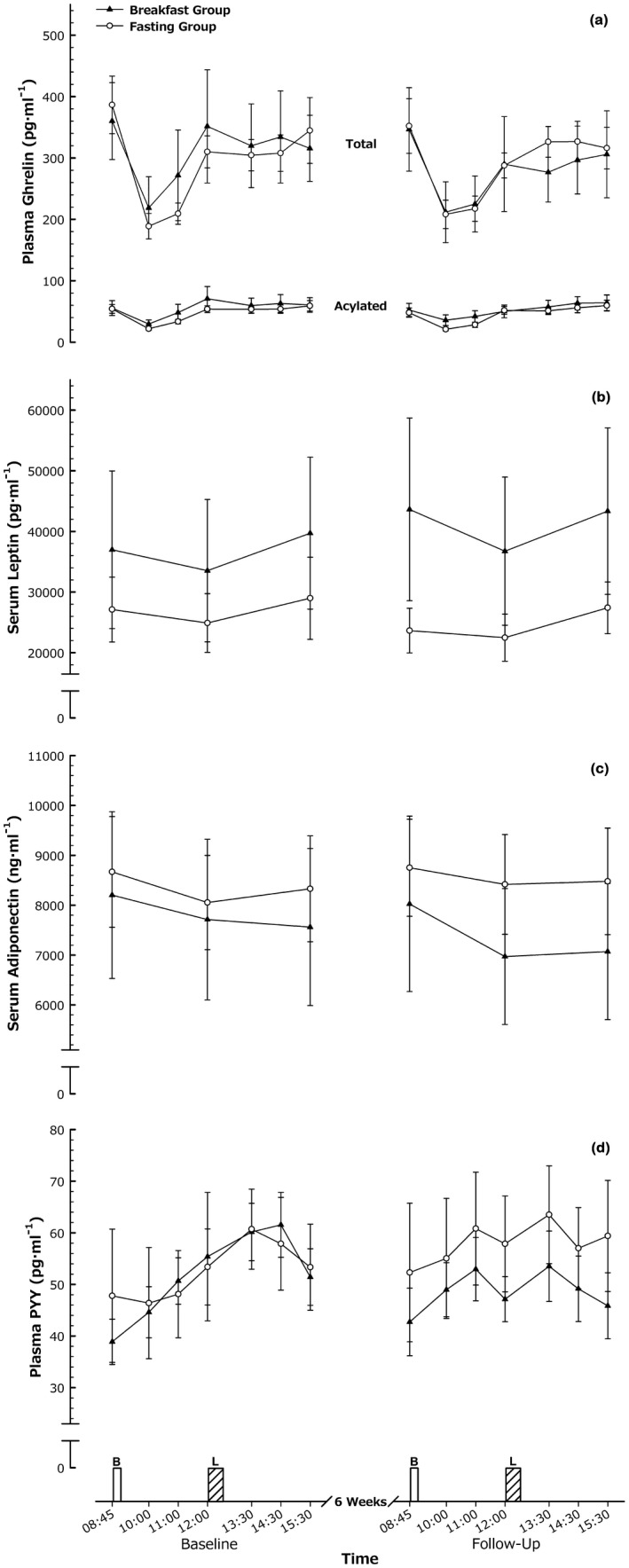
Energy balance regulatory hormones. (**A**) Plasma total and acylated ghrelin, (**B**) serum leptin, (**C**) serum adiponectin, and (**D**) plasma PYY responses to feeding in men and women with obesity before and after a 6‐week intervention. Values are mean ± SEM. B, breakfast; L, *ad libitum* lunch.

The time course of several hormones involved in the regulation of appetite and energy balance are illustrated in Figure 4. There were no group × trial interactions for either AUC or time course data for the regulatory hormones (all *P* > 0.10), apart from a tendency for time course data (*P* = 0.08) and AUC of PYY (*P* = 0.05) to indicate a reduction relative to the fasting group following the breakfast intervention. There was also a group × trial interaction for adiponectin AUC (*P* = 0.04) but not for time course (*P* = 0.10). For these measures, there were no main effects or interactions for the specific comparisons of peak, nadir, or fasting concentrations (all *P* > 0.11).

### Diet‐induced thermogenesis

**Figure 5:**
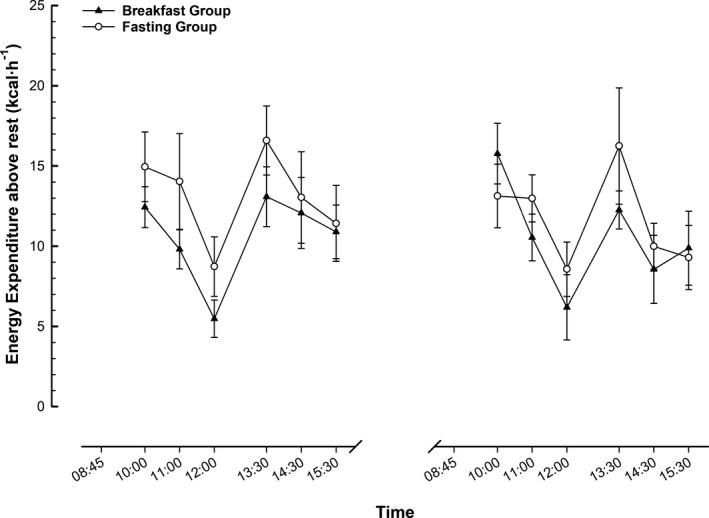
Diet‐induced thermogenesis. Energy expenditure above rest during feeding trials in men and women with obesity before and after a 6‐week intervention. Values are mean ± SEM. B, breakfast; L, *ad libitum* lunch.

There were no group × trial interactions for diet‐induced thermogenesis when separated for the morning or afternoon or when considered for the day overall (all *P* > 0.7; Figure 5). Diet‐induced thermogenesis measured over the day was 64 ± 21 kcal before the intervention and 63 ± 26 kcal after in the breakfast group, and it was 79 ± 36 kcal versus 70 ± 23 kcal in the fasting group.

### Appetite sensations

**Figure 6:**
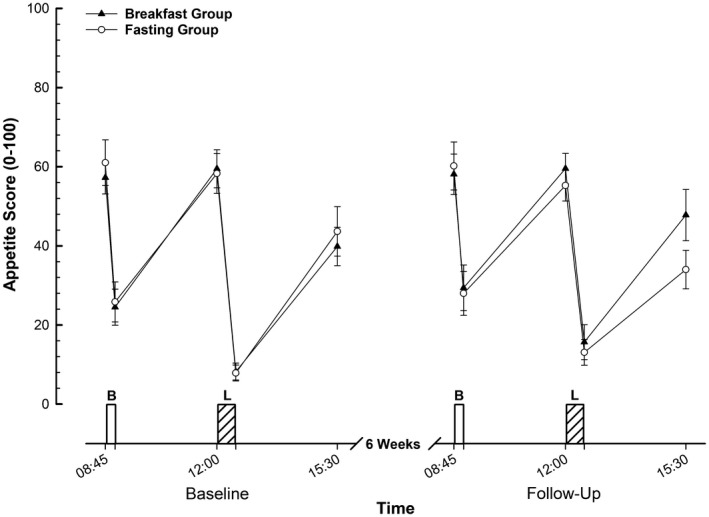
Composite appetite scores during feeding trials in men and women with obesity before and after a 6‐week intervention. Values are mean ± SEM. B, breakfast; L, *ad libitum* lunch.

Appetite varied over time, with a main effect of time (*P* < 0.01) for the composite appetite score displayed in Figure 6. There were no other main effects or interactions for either time course data or fasting appetite (all *P* > 0.1). For all of the subcomponents of appetite (desire to eat, hunger, fullness, and prospective consumption), there was a main effect of time (all *P* < 0.01) but no other main effects or interactions for time course data (all *P* > 0.1) apart from a group × trial interaction for hunger (*P* = 0.05). This response was characterized by greater hunger in the breakfast group at follow‐up, with less hunger in the fasting group. There were no main effects or interactions for fasting measures of any of the individual components of appetite (all *P* > 0.1).

## Discussion

The present study is the first to examine the extent of adaptation in acute metabolic and appetite responses following differing sustained morning feeding and fasting patterns in adults with obesity. Consistent with our hypothesis, there was no indication of altered postprandial insulin sensitivity in either group, and the vast majority of appetite hormone responses remained stable following the intervention, although PYY was reduced in the breakfast group. Following a standardized breakfast, there was no significant difference in *ad libitum* lunch intake based on group allocation during the interventions. Diet‐induced thermogenesis was not affected by either intervention. Subjective appetite responses were broadly similar but indicated greater perceptions of hunger following the breakfast intervention with reductions following daily fasting. Therefore, these data suggest that 6 weeks of extended morning fasting versus breakfast consumption does not meaningfully alter the acute energy intake, appetite, or metabolic responses to acute feeding in adults with obesity.

In agreement with our previous findings in lean individuals 18, appetite hormone responses throughout the day were not affected by either the daily breakfast or morning fasting regimen. This appears to highlight that despite several investigations identifying different concentrations or patterns of response of appetite hormones in individuals with obesity 20, 21, 22, 35, 36, 37, 38, these regulatory hormones do not exhibit any adaptation to differing daily morning feeding regimens over this time scale. The only appetite hormone affected by the intervention was PYY, which tended to be lower during the afternoon following the breakfast intervention. The degree to which this is potentially a product of the slightly reduced lunch intake at follow‐up observed in this group is unclear. However, evidence does not support lunch intake changes being an important factor, as correlation between the changes from baseline to follow‐up in lunch intake and peak afternoon PYY concentration was very weakly related (*R* = 0.23, *P* = 0.58). On the whole, our results reinforce previous evidence that meal pattern seems to have little impact on appetite hormone responses to feeding in the context of relative weight stability 39.

In a previous study, lean women presented with reduced insulin sensitivity in response to a 2‐week intervention in which the first feeding of the day was delayed until 11 am 40. This finding corresponds with some of our previous work in this cohort of individuals with obesity, with reduced insulin response to an oral glucose tolerance test in those consuming breakfast relative to those fasting during the 6‐week intervention 24. Our current investigation using a mixed meal does not support these findings, as insulin concentrations were unaffected by either intervention. This discrepancy could be due to recently reported differences in muscle microvascular perfusion with oral glucose ingestion relative to a mixed meal 41, which highlight that fat and protein may have played a role in the discrepancy between the methods as well as the importance of utilizing mixed‐macronutrient meal challenges for generalizability. It should also be considered that other related measurements such as homeostatic model assessment‐insulin resistance, insulin sensitivity indices, and adipose tissue glucose uptake in response to insulin were found to be unaffected by either intervention 24. Additionally, we determined little evidence of differential regulation of gene expression or protein content or activation of elements involved in adipose tissue insulin signaling in individuals with obesity in response to these interventions 23. Therefore, the balance of our work does not suggest a strong effect on insulin sensitivity and glycemic control, particularly within the most ecologically relevant setting of mixed‐meal ingestion. However, further work is required to substantiate the effects of morning feeding patterns on insulin sensitivity, including examination of molecular responses within muscle to provide additional mechanistic insight.

Prior work investigating meal patterns in women with obesity demonstrated that an irregular meal pattern can reduce diet‐induced thermogenesis 42. The authors suggested this was due to the reduced insulin sensitivity induced by the intervention and the established links between these phenomena 43. Therefore, as insulin sensitivity was not majorly affected in this cohort, it is unsurprising diet‐induced thermogenesis was also unaffected by either intervention in our current investigation.

In line with lean participants previously studied using the same experimental paradigm (for whom no adaptation in acute energy intake was observed) 18, there was no statistical evidence of differing energy intake during the *ad libitum* lunch following adherence to the daily breakfast intervention among this cohort with obesity. However, though not statistically significant for a group × trial interaction, there was evidence of slightly reduced lunch intake in the breakfast group from before to after the intervention. The reduction observed is somewhat surprising considering that there was greater reported subjective hunger prior to lunch following the daily breakfast regimen. Therefore, either the food provided at lunch became more satiating following the intervention (explaining the reduced intake), or this provides more evidence of a potential dissociation between subjective measures of appetite and objective measures of intake in individuals with obesity 44.

Similar to the results described for prelunch hunger ratings, for individuals who undertook the daily breakfast intervention, hunger was greater at the end of the testing day post intervention. However, as discussed, participants also consumed slightly less at the *ad libitum *lunch. Therefore, it is unclear whether this response was simply a product of slightly reduced energy intake at the *ad libitum *lunch in the breakfast group or meaningful adaptation of appetite sensations to chronic exposure to the intervention. One piece of evidence that would suggest that the difference in lunch intake is unlikely an explanation is that the relationship between difference in lunch intake and hunger from baseline to follow‐up was very weakly related in this group (*R* = −0.25, *P* = 0.47). An additional piece of evidence that may suggest a genuinely exacerbated return of hunger after eating in the breakfast group is that hunger was also increased 3 hours after the fixed breakfast meal post intervention.

While our current design provides valuable information regarding self‐selected intake, different designs would be required to establish the specific effects on subjective appetite of feeding interventions. Fixed lunch meals, as previously employed by Thomas et al. (17) when investigating the impact of self‐reported breakfast habits on metabolic responses to morning fasting or feeding, would be appropriate to remove this potential confounding factor and allow direct comparisons before and after interventions. Because of the integrated nature of this study within a broader project, the study was not specifically powered for the outcome variables reported herein, and it is possible that the study was underpowered to find potentially significant differences. Therefore, it would also be of interest to extend the length of the interventions beyond 6 weeks with greater participant numbers and to examine the effects of the prolonged interventions on acute responses to a day incorporating morning fasting.

The current study provides little evidence of adaptation in appetite regulatory hormone, metabolic, or energy intake responses to a fixed breakfast or *ad libitum *lunch following 6 weeks of daily breakfast or morning fasting in adults with obesity. Furthermore, the morning fasting intervention did not alter postprandial insulin or glucose responses or diet‐induced thermogenesis. In conclusion, this work suggests that most effects of morning fasting or feeding are likely to be transient. Acute responses to feeding do not appear to be altered with sustained changes in breakfast consumption habits, so discrepancies between self‐reported breakfast consumers and skippers may reflect wider differences in lifestyle.
